# Pathophysiological and Pharmaceutical Considerations for Enhancing the Control of *Sarcoptes scabiei* in Wombats Through Improved Transdermal Drug Delivery

**DOI:** 10.3389/fvets.2022.944578

**Published:** 2022-06-28

**Authors:** Jaskaran Bains, Scott Carver, Susan Hua

**Affiliations:** ^1^Therapeutic Targeting Research Group, School of Biomedical Sciences and Pharmacy, University of Newcastle, Callaghan, NSW, Australia; ^2^School of Natural Sciences, University of Tasmania, Hobart, TAS, Australia; ^3^Precision Medicine Research Program, Hunter Medical Research Institute, New Lambton Heights, NSW, Australia

**Keywords:** wombats, sarcoptic mange, transdermal drug delivery, moxidectin, fluralaner, ivermectin

## Abstract

*Sarcoptic scabiei* is an invasive parasitic mite that negatively impacts wombats, causing sarcoptic mange disease, characterized by alopecia, intense pruritus, hyperkeratosis, and eventual mortality. Evidence suggests that wombats may be unable to recovery from infection without the assistance of treatments. Transdermal drug delivery is considered the most ideal route of administration for *in situ* treatment in free-ranging wombats, as it is non-invasive and avoids the need to capture affected individuals. Although there are effective antiparasitic drugs available, an essential challenge is adequate administration of drugs and sufficient drug retention and absorption when delivered. This review will describe the implications of sarcoptic mange on the physiology of wombats as well as discuss the most widely used antiparasitic drugs to treat *S. scabiei* (ivermectin, moxidectin, and fluralaner). The prospects for improved absorption of these drugs will be addressed in the context of pathophysiological and pharmaceutical considerations influencing transdermal drug delivery in wombats with sarcoptic mange.

## Introduction

Sarcoptic mange, caused by the parasitic mite *Sarcoptes scabiei*, is among the most host-generalist and burdensome of parasitic diseases impacting mammals—wildlife, domestic animals, and humans (1–3). Research has shown the parasite to be documented in ~150 species, representing 12 Orders and 39 Families. In addition to impacts on domestic animals and wildlife, *S. scabiei* continues to cause significant human disease burden, with a point prevalence of ca. 100 million cases, remains among the 30 most prevalent infections of humans, and was recently recognized as a Neglected Tropical Disease by the World Health Organization (4, 5). Evidence suggests that the global distribution of *S. scabiei* is associated with European colonialism (both people and their domestic animals), leading to numerous spill over and pathogen establishment events (3, 6, 7).

*S. scabiei* mites infect the host by penetrating the stratum corneum, which is the outermost layer of the epidermis and functions as the main barrier of the skin. The mites are able to secrete a clear fluid to lyse the stratum corneum, thereby allowing them to dig into the depression (8) and reside between the stratum lucidum and stratum granulosum (9). Due to skin proliferation, the *S. scabiei* mites burrow toward the basal cell layer and dermis in order to maintain this location in the lower epidermis (8). The mites survive by ingesting extracellular fluid containing host immunoglobulins that seeps into the burrow from the lower vascular dermis (10). Mature female mites are able to reproduce and lay eggs at a rate of 2–3 per day (8, 9).

The main signs and symptoms of sarcoptic mange are attributed to an allergic reaction to the mites, resulting in intense pruritus, alopecia, hyperkeratosis, open wounds, infections, and emaciation (1). A range of antiparasitic drugs have been shown to be effective in controlling *S. scabiei* infection. However, the essential challenge of control of *S. scabiei* in host individuals and populations can be broken down into three key features: (i) having access to efficacious drugs; (ii) effective administration of drugs to impacted hosts; and (iii) adequate drug retention and absorption when delivered. The latter two factors require improvement for the treatment of sarcoptic mange in animals.

Topical drug delivery is considered the most ideal administration route for treating sarcoptic mange in free-ranging animals, as it is non-invasive and avoids the need to capture affected individuals (11). For antiparasitic drugs to be effective, they need to be delivered into the systemic circulation *via* transdermal absorption to provide adequate drug concentrations to treat and prevent the *S. scabiei* infection (12–14). Therefore, the goal for transdermal drug delivery is to transport the drug to the deeper dermis and subcutaneous tissues that are highly vascularized (15).

In this review, we focus on prospects for improved absorption of the three most widely used drugs to treat *S. scabiei* across all non-human host species—ivermectin, moxidectin, and fluralaner. This focus was chosen because it has received comparatively little attention in the scientific literature for this specific parasitic infection. These prospects will be discussed in the context of the wombat host, addressing both pathophysiological and pharmaceutical considerations, owing to the impact of *S. scabiei* on this marsupial and the fact that all three of the focal drugs are used to treat them for sarcoptic mange.

## Exposure, Transmission, Physiological and Behavioral Implications of *S. scabiei* Infection in Wombats

Sarcoptic mange is the most impactful disease of the bare-nosed wombat (*Vombatus ursinus*). This fossorial marsupial has a largely solitary lifestyle, and exposure to *S. scabiei* is generally thought to occur *via* environmental transmission (6). Bare-nosed wombats shift burrows every 4–10 days and, thus, share burrows asynchronously (16). Environmental transmission occurs when a mangy wombat deposits mites within a burrow environment and a newly colonizing wombat becomes exposed to those, likely in the bedding chamber. Evidence suggests *S. scabiei* mites may survive off-host within wombat burrows for 6–16 days depending on the time of year and, because this survival time can exceed burrow switching rates, environmental transmission can be sustained (17).

The intense irritation caused by *S. scabiei* infestation causes the wombats to scratch excessively as well as rub against objects (18). Hyperkeratosis associated with the host's immunopathological response and chronic scratching can cause thickened skin to fissure, become flyblown and lead to secondary infections (local and systemic), which are usually fatal (18–20). Disease progression leads to elevated metabolic demands, impaired mobility and foraging performance, and loss of body condition (18, 21). Without effective treatment, infection is considered to progress with death usually resulting from secondary infection and/or starvation (18, 20). Disease severity in wombats is attributable to a type IV hypersensitivity reaction (22), whereby the proliferation of *S. scabiei* mites on the host is largely unregulated by the host's immune response (22).

Rose and Higgins (23) reported the details of an autopsy that was performed on a bare-nosed wombat with severe sarcoptic mange infection, focusing on the tissue integrity in the dorsal, ventral, rump, and facial regions. The wombat exhibited severe alopecia and hyperkeratosis, characterized by thickened proliferative white crusts with numerous cavernous chambers that penetrated 1 cm deep into the dermis. Histological analysis demonstrated that the chambers were flooded with proteinaceous fluid and erythrocytes harboring numerous bacterial colonies. Further investigations showed that the wombat's skin displayed signs of dehydration, with emaciated muscle mass and absent fat deposits. The findings of this detailed case is consistent with other reports in the literature which also found the wombats to be in poor physiological condition, with swollen lymph nodes and lacking subcutaneous fat (24). Skerratt et al. (24) reported that the pathological changes were consistent with anemia, inflammation, and starvation owing to disease progression.

Behavioral changes have also been reported in wombats as the severity of the mange increases. Key behavioral changes include: increased foraging times and activity during diurnal periods; greater home range size compared to healthy wombats; increases in burrow visitation and switching; and increased scratching behaviors (18, 21). As disease progression also leads to sensory deprivation in wombats (21), they can also become more approachable to administer treatments (25).

## Current Therapeutic Approaches to Treat Sarcoptic Mange in Wombats

Early diagnosis and treatment of sarcoptic mange in wombats rarely happens. By the time clinical signs are apparent in wombats (e.g., hyperkeratosis and alopecia), they are at a comparatively moderate-to-advanced state of the disease. Several treatment strategies have been proposed and/or trialed. The strategy with the highest success rate is to capture the wombats and directly administer the antiparasitic medication topically and/or systemically (*via* injection) (13). However, there are several problems with this method: (i) difficulty in capturing as well as placing the wombats in captivity increases their stress levels which can adversely affect the animals (26); (ii) if the treatment is successful and they are released back into the wild, the wombats would likely return to the same mite-infested environments (11); and (iii) there are limited number of rehabilitators that are capable of successfully capturing, housing, and rehabilitating mange infected wombats. Therefore, most animals are treated *in situ* in their natural environment, using either direct topical application of the medication (e.g., “pole and scoop” method) to essentially pour the topical medication onto the wombat's upper back from a distance, or indirect topical application (e.g., remote “burrow-flap” stations) whereby antiparasitic medications are linked to mechanisms that trigger release onto the dorsal region of the wombat as they enter or leave the burrow (26). There are obvious difficulties with these approaches, including identifying specific wombats for treatments requiring multiple doses and inadequate drug absorption following topical administration with the available antiparasitic treatments. The outcome of most treatments is unknown and treatment failure is most probably common. In practice, *in situ* treatment generally fail due to “run off” of the formulation after it is poured onto the wombat and poor drug penetration through the fur and hyperkeratosed skin barriers. Overcoming these challenges will provide significant advantages for the treatment of this parasitic disease not only in wombats but also for other animals with sarcoptic mange in the wild.

## Pathophysiological Considerations Influencing Topical Drug Delivery in Wombats

Pathophysiological factors can significantly affect transdermal drug delivery of a formulation (27). Factors such as tissue integrity, dermal inflammation, and metabolic demand can lead to variations in drug absorption following topical administration and, therefore, can affect bioavailability, efficacy, and safety of the compound. Sarcoptic mange affects the integrity and quality of the skin of wombats (23) in a way that drug absorption can be compromised. This section will discuss the main pathophysiological barriers to effective transdermal drug delivery in wombats with sarcoptic mange, which should be considered in the rational design and development of pharmaceutical formulations.

### Fur Covering the Skin

The most obvious barrier to effective topical drug delivery, especially using the pour-on approach for drug administration, is the fur coat that covers the skin of wombats. In order for drug absorption to occur across the skin for dermal or transdermal drug delivery, the formulation needs to be in direct contact with the skin for an adequate period of time (12). Therefore, the thick and coarse fur coat of wombats will likely act as a barrier and prevent some or all of the formulation from reaching the skin surface. In addition, environmental components adhering to the fur coat may also pose a barrier to effective topical drug delivery, such as dirt and soil. To bypass this barrier, the physicochemical properties of the formulation will need to minimize binding to the fur coat and enhance movement toward the skin surface. The increased hair follicles may potentially allow enhanced drug delivery through the transappendageal route, which involves the dissemination of drug *via* the hair follicles and sweat glands (28, 29).

### Hyperkeratosis and Crusting of the Skin

In the bare-nosed wombat species, the dorsal epidermis was reported to lack a granular layer beneath the corneous layer and had an almost linear basal layer consisting of cuboidal cells overlain by flattened spinous cells as well as a thick stratum corneum (30). As sarcoptic mange infestation progresses in wombats, typical signs of chronic inflammation, hyperkeratosis and crusting of the skin occurs, which often leads to alopecia in those regions. Although the fur no longer acts as a main barrier in these cases, the thickened and crusted skin become a more prominent obstacle to effective topical drug delivery.

*S. scabiei* mites have been reported to trigger epidermal stimulation and increase production of keratinocytes resulting in the epidermal thickening after a day post-infection (22). This early stage of inflammation may enhance drug absorption by causing intercellular dilation, which augments epithelial permeability (22); however, under chronic inflammatory conditions leading to hyperkeratosis of the skin, this effect is diminished. As a result, free drug molecules have difficulty permeating through the wombat skin in significant amounts, especially during moderate-to-severe sarcoptic mange infections, likely leading to reduced therapeutic efficacy and incomplete recovery (14, 26). Higher doses have been administered in an attempt to provide adequate systemic drug concentrations following transdermal drug delivery in an already more compromised animal (31). However, administering higher doses does not always equate to increased efficacy and can instead increase the risk of adverse effects such as skin irritations and dermal toxicity. For completeness, it should be noted that drug absorption is usually higher at sites where there are open wounds. Open wounds commonly occur in mange infested wombats due to excessive and aggressive scratching.

### pH of the Skin

While the skin pH of wombats has not been extensively studied, it is a factor that can affect topical drug delivery. For formulations containing free drug molecules, the ionization state of the drug will impact its ability to permeate the skin barrier. The drug ionization state is affected by the pH of the skin, the pH of the formulation base, and partition coefficient of the drug (12). Unionized compound are able to permeate through lipophilic regions at higher therapeutic rates compared to ionized drugs (32). However, for effective transdermal drug delivery there needs to be a balance between both lipophilic and hydrophilic properties to enable passage across the skin barrier and cell membranes as well as through aqueous pathways in the epidermis and dermis of the skin (33, 34). In addition, formulations should have a pH as similar to the normal skin pH of the target species, as deviations can lead to skin irritation and other adverse effects to the skin (12).

### Moisture Content of the Skin

Skin hydration is important to maintain the barrier function of the stratum corneum, enzymatic activity, and normal cell division and differentiation (15, 34). In general, increased skin hydration leads to increased topical drug absorption. However, in wombats suffering from sarcopic mange, reduced skin hydration has been reported due to chronic inflammation, hyperkeratosis, and crusting of the skin (23, 35). Therefore, modifications in the moisture content and/or occlusive nature of the pharmaceutical formulation may assist to enhance drug absorption across the skin (15, 34).

### Skin Vascularity

For effective transdermal drug delivery, considerations should be made to not only skin penetration of the formulation but also permeability to the deeper layers of the skin (36). The rate of drug clearance from the skin is influenced by vascular parameters including thickness of local and surrounding blood vessels, blood flow rate, and distance between blood vessels (12). Drug delivery to the dermis and hypodermis (subcutaneous tissue) is advantageous for transdermal drug delivery owing to the larger size and number of blood vessels in those deeper skin layers. Similarly, topical administration to areas of high vascularity would be another means to improve transdermal drug delivery. The current site for topical administration of antiparasitic drugs, especially for *in situ* treatment of wombats with sarcoptic mange, is the dorsal region of the back. This site has been used for practical reasons and is easier to access compared to other more vascularized regions (e.g., underbelly of the wombat) (30).

### Metabolic Demands

The chronic inflammation associated with sarcoptic mange contributes to the increased metabolic demands in wombats, thereby reducing fat stores and altering fatty acid composition, with increased amounts of omega-6 and decreases in omega-3 levels reported (21). As the severity of sarcoptic mange increases, so too does the metabolic demand (18). This can particularly affect lipophilic medications that accumulate in fat stores to create a “depot-like” effect for sustained drug release. Reduced fat stores may decrease the half-life and duration of action of these specific drugs.

## Comparison of the Main Pharmacological Therapies to Treat Sarcoptic Mange in Wombats

Pharmacological interventions are still the mainstay of treatment for wombats affected with sarcoptic mange. The goal is to treat the *S. scabiei* infection and prevent reinfection over a prolonged duration, which will also result in the death of environmental fomites. This section will provide a summary of the mechanism of action, physicochemical properties, pharmacokinetics, efficacy, and safety of the main antiparasitic drugs that have been used to treat *S. scabiei* infections—ivermectin, moxidectin, and fluralaner (Table 1). These characteristics are important to understand, especially for pharmaceutical scientists, in order to improve rational formulation design for transdermal drug delivery for clinical use.

**Table 1:** Summary of physicochemical, pharmacokinetic and pharmacodynamic parameters for current treatments of sarcoptic mange in wombats.

**Drug properties**	**Ivermectin**	**Moxidectin**	**Fluralaner**
Chemical structure	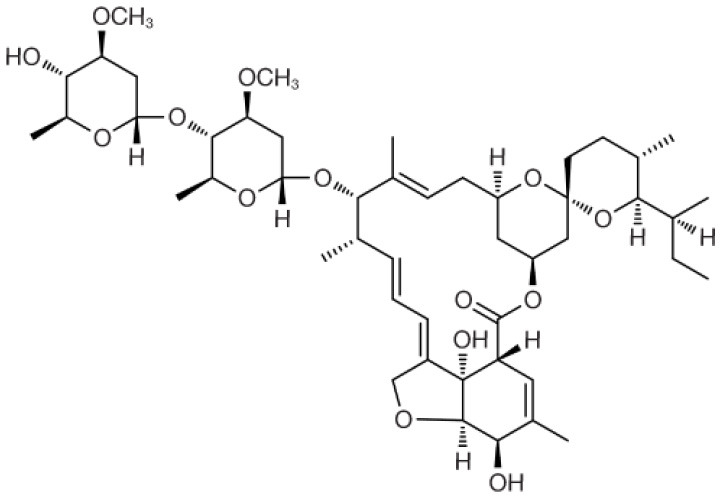	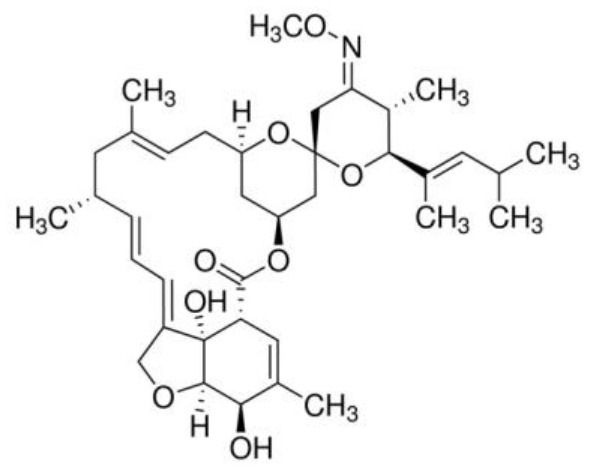	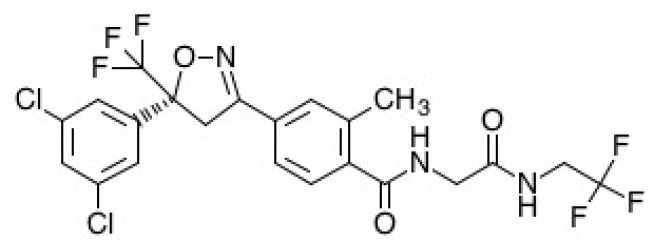
Molecular formula	C_48_H_74_O_14_	C_37_H_53_NO_8_	C_22_H_17_Cl_2_F_6_N_3_O_3_
Molecular weight	875.1	639.8	556.29
Log partition coefficient (octanol/water)	4.8	6	5.35
pKa	12.47	12.8	12.50
Half-Life at recommended dose	~48.5 h (42)	~5 days (50)	~40 days (14)
Drug class	Avermectin (37)	Milbemycin (37)	Isoxazoline (56)
Antiparasitic spectrum	Broad (37)	Broad (37)	Broad (56)
Mechanism of action	Binds to GABA and glutamate-gated chloride channels in parasites. This binding action results in an influx of chloride ions causing paralysis in the parasite, eventually leading to its death. Also has high affinity to P-glycoprotein (38)	Binds to GABA and glutamate-gated chloride channels in parasites. This binding action results in an influx of chloride ions causing paralysis in the parasite, eventually leading to its death (48)	Inhibits GABA and L-glutamate-gated chloride channels resulting in the paralysis and death of the mites (57)
Ability to kill eggs	No (38)	No (48)	Yes, after hatching (57)
Recommended dose	Subcutaneous injection of 0.2–0.4 mg/kg weekly over several months (40)	Maximum 20 ml (0.8 ml/kg) applied topically once a week, and repeated weekly for 15 weeks (31)	One application of 25 mg/kg applied topically (14)
Efficacy	Somewhat efficacious (20, 44)	Efficacious (13, 26, 50, 51)	Efficacious (14)
Safety	Safe at standard dose and relative safety at high doses (19, 37)	Safe at standard dose and relative safety at high doses (52–55)	Safe in standard and high doses (14, 58–60)
Protection period	<10 days (20)	2–10 days (50)	4–15 weeks (14)

*Physicochemical properties attained from PubChem (pubchem.ncbi.nlm.nih.gov)*.

### Ivermectin

Ivermectin is a first-generation macrocyclic lactone of the avermectin class (37). The hydrophobic macrocyclic lactone ring allows the drug to bind to GABA and glutamate-gated chloride channels in parasites, thereby resulting in an influx of chloride ions causing paralysis and death of the parasite (38). Ivermectin also binds to P-glycoprotein, which acts as permeability barrier for the passage of molecules across cell membranes (38). This may contribute to the low biodistribution of ivermectin in specific tissues of the host (38) and can also affect penetration into parasites that have developed resistance using P-glycoprotein mechanisms (39).

The main characteristics of ivermectin have been summarized in Table 1. Although effective as an antiparasitic compound, there are several limiting factors for the use of ivermectin for the treatment of sarcoptic mange in wombats. Ivermectin is usually administered *via* subcutaneous injection weekly over several months (40), which is not the ideal administration route for *in situ* treatment in free-ranging wombats. The drug also provides an antiparasitic protection period of <10 days post-dose (20). This may not be adequate for prolonged protection from the ongoing mite deposition in burrows, potentially leading to higher susceptibility for reinfection (13). Additionally, there appears to be an emerging resistance to ivermectin in *S. scabiei* (41).

Understanding the pharmacokinetics of a drug is important for both clinical practice and pharmaceutical development, as it informs how a compound is processed within the body (i.e., absorption, distribution, metabolism, and excretion) and how this can be modified in rational drug and formulation design. It should be noted, however, that there are no pharmacokinetics studies available in wombats to date. Data collected from ivermectin use in other mammals may help to inform the potential pharmacokinetics of this drug in wombats—although physiological considerations will need to be applied across species when interpreting the data. In livestock, intravenous injection had a general half-life of 32–65 h (42). The half-life of a drug informs the time taken for the concentration of a drug in the plasma or the total amount in the body to be reduced by 50%. Following subcutaneous injection, the drug was thought to be distributed more into lipid reservoirs in the skin tissue as there was a residence time of 114–216 h, which was three times longer compared to intravenous administration (42). Drug accumulation in adipose tissue can lead to prolonged drug release into the systemic circulation. In terms of elimination, ivermectin was only partly metabolized following subcutaneous administration, with a considerable amount of the parent drug excreted in feces (43). This was suggested to have negative consequences on nutrient cycling in the ecosystem (43).

There are a number of studies available supporting the effectiveness of ivermectin as an antiparasitic compound against *S. scabiei* in wombats (20, 44). Standard doses have also been shown to be safe in mammals due to higher specificity of glutamate-gated chloride channels which is only present in parasites (37). In wombats, there was no reported side effects from an observational study other than higher incidences of recurrence from mange (19). Although the safety of ivermectin on wombat reproduction is unknown, mammalian studies have shown ivermectin to be safe in pregnancy (45, 46). However, owing to its lipophilicity, ivermectin can concentrate in the mother's milk and this should be considered for suckling offspring (46). Furthermore, ivermectin has been shown to decrease sperm count and increase sperm abnormalities in male rats (47). As rodent studies are commonly used to inform safety and efficacy of therapeutic compounds for potential human use, this should also be taken into account for other non-human uses.

### Moxidectin

Moxidectin has highly potent endectocide activity (37). It is a potent second-generation macrocyclic lactone of the milbemycin class (37) and has a similar mechanism of action as ivermectin (48). However, moxidectin is a poor substrate for P-glycoprotein, which decreases the risk of CNS side effects and is less susceptible to elimination from parasites (49). In addition, moxidectin is more lipophilic compared to ivermectin, owing to the methoxime moiety at carbon-23 (48).

Moxidectin is the most used antiparasitic drug for the treatment of wombats with sarcoptic mange. For this indication, it is usually applied topically as a pour-on treatment. The optimal dose and dosing frequency are still areas of contention, with multiple recommended dosing regimens used over the years (31). For example, Table 1 shows one of the recommended weekly application regimens that has a treatment duration of several months. Moxidectin has been reported to provide an antiparasitic protection period of between 2 and 10 days post-dose (50), which like ivermectin, may not be adequate for prolonged protection from the ongoing mite deposition in burrows.

With regards to pharmacokinetic parameters, there is only one pharmacokinetic study of moxidectin conducted in wombats. Moxidectin was reported to have a half-life of 5 days in wombats following both subcutaneous injection and topical application (50)—hence, it has a longer duration of action compared to ivermectin. Although not specifically in wombats, moxidectin has been shown to distribute to adipose tissue following administration in horses (48), which is likely due to its lipophilic nature. Therefore, it would be expected for moxidectin to accumulate in the hypodermis (subcutaneous layer) following topical administration to some degree, as this layer generally contains more adipose tissue compared to the other skin layers. Moxidectin was found to be primarily excreted in the feces and very little in urine across various routes of administration (subcutaneous, oral, and topical) (42).

There are a number of studies available supporting the effectiveness of moxidectin as an antiparasitic compound against *S. scabiei* in wombats (13, 26, 50, 51). It should be highlighted, however, that there is variation in the outcome of treatment among individual wombats and, in most cases, the outcome is not known with *in situ* treatment. Potential reasons for treatment failure include the baseline degree of sarcoptic infection in individual wombats, inefficacy of eliminating mite ova resulting in reinfection, and issues with efficient drug delivery following topical application. The therapeutic index (i.e., margin of safety that exists between the dose of a drug that produces the therapeutic effect and the dose that produces toxicity) for moxidectin is relatively unknown in wombats. Data from other mammalian species have shown a wide margin of safety (52–55).

### Fluralaner

Fluralaner is an isoxazoline that acts as a second-generation GABAergic compound (56). It inhibits GABA and L-glutamate-gated chloride channels in mites, which causes death by paralysis (57). Fluralaner is best known as an antiparasitic treatment product for dogs and cats, commercially available as Bravecto^®^. It has only recently been investigated for use in wombats with sarcoptic mange (14). Fluralaner is dosed based on the size of the wombat (Table 1) and is administered as a single spot-on treatment that is applied directly to the surface of the skin.

Wilkinson et al. (14) were the first to investigate the pharmacokinetics, efficacy and safety of fluralaner in bare-nosed wombats with sarcoptic mange. Fluralaner doses of 25 and 85 mg/kg in wombats resulted in a Cmax (maximum plasma concentration) of 6.2 and 26.4 ng/ml with a Tmax (time to maximum plasma concentration) of 3 and 37.5 days, respectively (14). This was followed by a half-life 40.1 and 166.5 days, AUC (area under the curve) of 152.9 and 516.8 ng.day.ml^−1^, and MRT (mean retention time) of 32 and 46.8 days, respectively (14). These pharmacokinetic data indicate significantly longer duration of action for fluralaner following a single dose compared to ivermectin and moxidectin. This is particularly advantageous for *in situ* treatment of sarcoptic mange in wombats, whereby treatments that require multiple dosing can be challenging. Although not yet investigated in wombats, fluralaner was reported to be highly bound to plasma proteins in dogs (57), which may also contribute to its longer duration of action.

Fluralaner has shown significant potential as a novel treatment for sarcoptic mange in wombats. Wilkinson et al. (14) reported that wombats in captivity with mild mange symptoms showed 100% recovery after 3 weeks post-dose with a single spot-on treatment, and those with moderate symptoms showed complete recovery after 4 weeks. The single dose spot-on solution was suggested to provide protection for up to 15 weeks against parasitic infection. This was inferred based on the wombats not acquiring other ectoparasites (e.g., ticks) by that time point in an outdoor enclosure (14). The long duration of action of fluralaner has the benefit of being able to eliminate hatchlings from *S. scabiei* mite ova, which has been a limitation of the other antiparasitic drugs. The study also suggested a potential pour-on treatment made by mixing commercially available spot-on treatment (Bravecto^®^) with an additional 5 ml of Orange Power Sticky Spot and Goo Dissolver^®^ (contains ethanol, D-Limonene, glycol ether, alcohols ethoxylated, citrus terpenes in orange peel oil) (14). This pour-on formulation would be better suited for *in situ* treatment in free-ranging wombats using the “pole and scoop” method of delivery; therefore, evaluation in wombats would be warranted.

The clinical safety information for fluralaner is much greater compared to the other antiparasitic compounds. As mentioned previously, fluralaner has been extensively used as an antiparasitic treatment for dogs and cats without causing any significant adverse effects (58–60). Similarly, an initial study in wombats using a standard dose of 25 mg/kg and a high dose of 85 mg/kg (3.4 times higher than the standard dose in canines) showed fluralaner to be both safe and well-tolerated (14).

## Pharmaceutical Considerations and Future Directions for Improving Transdermal Delivery of Pharmaceuticals in Wombats

Transdermal drug delivery is challenging. The main factors that affect transdermal drug delivery are the physicochemical characteristics of the drug, the physicochemical characteristics of the formulation vehicle, and the physical state of the skin. As stated in the aforementioned section, the physical changes that occur to the skin in mange infested wombats significantly compromises topical drug absorption. These physical changes are progressive, therefore modification of the drug and/or formulation vehicle are the most feasible strategies to improve transdermal drug delivery.

The main difficulty of transdermal delivery is getting enough drug to penetrate through the skin, especially the stratum corneum (15, 61). The stratum corneum is the major rate-limiting barrier to transdermal drug transport (15, 61). Being a keratinized tissue, it behaves as a semipermeable membrane and drug molecules penetrate this barrier mainly by passive diffusion (15). However, not all drugs can be delivered by the transdermal route of administration due to the natural limits of drug penetration imposed by the skin's barrier function. In general, small drug molecules with good lipophilicity and adequate aqueous solubility are the ideal drug characteristics for transdermal drug delivery for passive absorption (32, 62). More specifically, the ideal drug candidate should have: (i) a molecular weight of <500 daltons; (ii) a log partition coefficient (octanol/water) between 1 and 4; and (iii) an aqueous solubility in excess of 100 μg/ml (33, 63). The dose required should be low at <20 mg/day (depending on the available surface area for administration) and the melting point of the drug should be <200°C (33, 62). Appropriate “flux” (i.e., the driving force for skin permeation) should be obtained for the drug, which is also dependent on the formulation vehicle (15). Furthermore, the free drug molecules should be in the unionized state for optimal permeability. Based on this information, it can be seen that the main antiparasitic drugs (ivermectin, moxidectin, and fluralaner) do not possess all the ideal physicochemical characteristics for transdermal drug delivery (Table 1). Modifying the chemical structure of drug molecules to achieve certain physicochemical characteristics may be considered; however, this is far more complex and can also adversely affect other properties of the compound such as efficacy and safety.

Instead, the formulation vehicle can be modified to enhance drug penetration across the skin barrier and allow adequate drug release kinetics. There are several mechanisms that can be utilized in formulation vehicle design to improve transdermal drug delivery: (i) altering the physicochemical properties of the stratum corneum; (ii) changing the hydration property of the stratum corneum; or (iii) altering the structure of proteins and lipids in the intercellular channel using formulation vehicle properties (33, 34). For application in wombats with sarcoptic mange, the formulation vehicle should also not adhere to the coarse fur coat but be able to move through this barrier to reach the skin. This is particularly important for effective drug delivery in free-ranging animals for *in situ* treatment that can be directly (e.g., “pole and scoop” method) or indirectly (e.g., burrow-flap stations) poured onto the animals. Direct contact and retention of the formulation to the surface of the skin would assist in maximizing topical drug absorption.

For antiparasitic treatments that can be effectively poured onto mange infected wombats, a liquid formulation vehicle (e.g., solution, suspension) is likely preferred over a semi-solid dosage form (e.g., cream, gel, paste). The latter would generally require capturing the animals and applying the treatment directly onto the skin surface. The liquid formulation can be adapted to enhance skin retention by increasing the viscosity of the formulation with lower percentages of polymers such as Carbopol^®^, hydroxypropyl methylcellulose (HPMC), or hydroxyethyl cellulose (HEC). In addition, chemical penetration enhancers can be added to the liquid formulation to enhance skin permeation of the drug molecules. These compounds can reversibly damage or alter the physicochemical nature of the stratum corneum to reduce its diffusional resistance (61, 64). It should be noted that the selection of permeation enhancer needs to be tested not only for efficacy in enhancing drug permeation across the skin, but also on its dermal toxicity and its compatibility with other components of the formulation. This is important as most would not have been investigated for use in wombats.

Similarly, other novel formulation strategies have been investigated to enhance transdermal drug delivery such as the use of nanoparticles (65). Although these have mainly been investigated for human use, they could potentially be applied for use in animals. Nanoparticles have the potential to significantly overcome the barrier of the stratum corneum due to their unique physicochemical characteristics, including size, composition, and surface properties (65). Nanoparticles may improve transdermal drug delivery by the following mechanisms: (i) penetration of intact nanoparticles into the different layers of the skin; (ii) interaction between nanoparticles with the stratum corneum thereby allowing more directed drug exchange; (iii) nanoparticle-mediated enhanced transdermal drug delivery *via* appendageal pathways (e.g., hair follicles and sweat ducts), and (iv) modifying the composition of nanoparticles with penetration enhancers to alter the skin barrier (65).

In addition to efficacy and safety, other considerations for improved topical formulations for the treatment of sarcoptic mange in wombats include ensuring structural and chemical stability during storage as well as having storage requirements that are suitable for dosing animals in the wild (e.g., not heat labile and does not require refrigeration). Finally from a translational point of view, simplification of the drug formulation design is required to allow efficient and reliable large-scale manufacturing for regulatory purposes. The formulation would also need to be cost effective and show significant therapeutic advantage over existing therapeutic strategies.

## Conclusion

Sarcoptic mange is a major disease of Australian wildlife and, in addition to being an individual welfare issue, has led to significant declines in the wombat population across some regions in Australia (21, 35). Without effective treatment, *S. scabiei* infested wombats rarely survive, with death usually resulting from secondary infection and/or starvation. Although the antiparasitic drugs are effective, the likely issue is poor topical retention and absorption of the current formulations in wombats, especially using a pour-on method to the dorsal region of the wombats' back. Topical drug absorption is made further challenging with the pathophysiological barriers that occur in sarcoptic mange infection in wombats. Developing improved antiparasitic formulations to treat sarcoptic mange in wombats in the wild will help in the conservation of these species that are native to Australia. *S. scabiei* has also been reported to affect a diverse host range in the environment, including domestic animals (e.g., dogs and cats), livestock (e.g., cattle, pigs and goats), and wildlife (e.g., koalas, wallabies, foxes, bears, coyotes, wolves, deer, and rabbits) (3). Therefore, developing improved antiparasitic formulations to combat this parasitic infestation will have important implications at a global scale for wildlife conservation and animal health, whereby animals do not need to be captured and held in captivity for sarcoptic mange treatment and, potentially, for the treatment of other infectious diseases.

## Author Contributions

SH was involved in conception of the idea for the review. All authors were equally involved in drafting the manuscript, revising the manuscript critically for important intellectual content, and approved the final version of the manuscript.

## Conflict of Interest

The authors declare that the research was conducted in the absence of any commercial or financial relationships that could be construed as a potential conflict of interest.

## Publisher's Note

All claims expressed in this article are solely those of the authors and do not necessarily represent those of their affiliated organizations, or those of the publisher, the editors and the reviewers. Any product that may be evaluated in this article, or claim that may be made by its manufacturer, is not guaranteed or endorsed by the publisher.
